# Rival perspectives in health technology assessment and other economic evaluations for investing in global and national health. Who decides? Who pays?

**DOI:** 10.12688/f1000research.13284.1

**Published:** 2018-01-17

**Authors:** Anthony Culyer, Kalipso Chalkidou, Yot Teerawattananon, Benjarin Santatiwongchai

**Affiliations:** 1 Department of Economics & Related Studies, University of York, UK, York, YO10 5DD, UK; 2 Global Health and Development Group, Institute of Global Health Innovation, Imperial College London, London, SW7 2AZ, UK; 3 Center for Global Development, Westminster Impact Hub, London, SW1Y 4TE, UK; 4 Department of Health, Ministry of Public Health, Thailand, Nonthaburi, 11000, Thailand

**Keywords:** HTA, CEA, perspective, stakeholder, social value judgments, reference case, patients’ preferences.

## Abstract

There seems to be a general agreement amongst practitioners of economic evaluations, including Health Technology Assessment, that the explicit statement of a perspective is a necessary element in designing and reporting research. Moreover, there seems also to be a general presumption that the ideal perspective is “societal”. In this paper we endorse the first principle but dissent from the second. A review of recommended perspectives is presented. The societal perspective is frequently not the one recommended. The societal perspective is shown to be less comprehensive than is commonly supposed, is inappropriate in many contexts and, in any case, is in general not a perspective to be determined independently of the context of a decision problem. Moreover, the selection of a perspective, societal or otherwise, is not the prerogative of analysts.

## Introduction

It seems a desirable thing to set international quality standards for conducting health technology assessments and other economic evaluations (EEs) of the impact and desirability of investments that promote health ^1^. Such standards are, however, not easy to define, let alone to get agreement on ^2^. What *is* easy is to fall into a trap of small-town thinking, by setting standards whose applicability is arbitrarily and quite needlessly restricted to: (a) particular cultural and political contexts, (b) within any such context, a set of choices bounded by further arbitrary value judgments, (c) a presumption that EE methods are designed for use only in the public sector – and, moreover, only when the object is “the public interest”. Parochialism has two unattractive features. First, it denies the possible usefulness of EEs outside the public sector and to non-governmental agencies, like trade unions, for-profit companies and non-profit organisations like charities. Second, it imports value judgments to the effect that (a) EE can be conducted in a context-free way without attention being given to cultural and political values that may be particular to the application, which may not be readily transferable from jurisdiction to jurisdiction and (b) that the use of patients’ (or the public’s) preferences, in constructing outcome measure such as the quality-adjusted life year (QALY) or disability-adjusted life year (DALY) via estimated or expressed consumer values, rather than using, say, judgments made by parliamentary committees, expert groups or citizens’ juries, provides an appropriate and relevant index of value. We seek to avoid such restraints by proposing that questions of perspective be determined in context, where the job of EE analysts is to help decision makers to articulate a perspective that is appropriate to the circumstances rather than to prescribe a perspective independently of its context of use (being “context free”).

First, we review the recent history of “perspective”.

## What is perspective and why does it matter?

Guides to best practice in cost-effectiveness analysis (CEA) can be traced back at least as far as 1980, with the publication, at the initiative of Senate Committee on Labor and Human Resources and commissioned by the then Office of Technology Assessment (OTA), of *The Implications of Cost-Effectiveness Analysis of Medical Technology* ^3^. Other significant landmarks appeared in 1994 by the Canadian Coordinating Office for Health Technology Assessment ^4^, in 1996 by Gold *et al*. (the “Washington Panel”) ^5^, in 1997 by Drummond *et al*. ^6^, in 2003 by the World Health Organization ^7^, in 2004 by the National Institute for Clinical Excellence (NICE) ^8^ and in 2016 by the second Panel ^9^ and the International Decision Support Initiative (IDSI) ^10^.

The Washington Panel coined the term “Reference Case” to describe the characteristics of a high quality economic evaluation, thereby establishing both a lasting piece of terminology and launching a stream of literature (see original publication here: https://www.ncbi.nlm.nih.gov/pubmed/8861994 and over 170 references in PubMed: https://www.ncbi.nlm.nih.gov/pubmed?LinkName=pubmed_pubmed_citedin&from_uid=8861994). The Panel’s work absorbed much of that by Drummond *et al* ^6^. One of the characteristics of a high-quality EE was the explicit stating of a study’s “perspective”, described by the panel as “the study’s point of view”, which “determines which health outcomes and costs are relevant and plays a part as well in how they should be valued”. We wholly agree with this principle and it seems to have formed a part of all subsequent reference cases, including one developed by the Drummond *et al*. team and us, through the Gates-sponsored iDSI Reference Case ( http://www.idsihealth.org/resource-items/idsi-reference-case-for-economic-evaluation/). The case for it is made not merely on grounds of transparency, which matters because it enables other researchers to check that the scope and measures of costs and benefits were indeed fit for purpose, but also on grounds of accountability: what to include and how to measure it are essential *political* and *social value* judgments that ought to be made by duly accountable decision makers (not EE analysts!). A good study will be, therefore, explicit about its perspective (or perspectives, if more than one is to be explored).

The Washington Panel recognised that a perspective must reflect the purpose of a study. It is not always appreciated that the perspective will usually be context-dependent, with the context determining the appropriate perspective. For example, a study might appraise the costs and benefits of a workplace intervention to reduce back strain on workers through redesigned work stations from the points of view of managers on the one hand and trade unions on the other, with a view to anticipating opposition and identifying compensatory and regulatory measures for successful implementation. In such a case, there are three perspectives to be considered – those of management, workers and government. The perspectives to be used are defined by the purpose of the study which in turn is specified by its sponsor: in this example, management, workers or the government (or a consortium of all three).

## When it comes to perspectives, is broader better?

The First Washington Panel’s Reference Case recommended that a societal perspective be adopted, offering decision makers as wide as possible an understanding of the costs and consequences of alternative actions both in the health care sector and beyond it into the private health care sector and non-health sectors, public or private. Its reasoning is plain: only if the societal approach is adopted can decision makers be confronted with a full information set of the costs and consequences of alternative actions. Anything less comprehensive will necessarily be subject to omitted variable bias, probably of unknown sign and size, causing either over- or under-investment in both old and new technologies. It is hard to raise an objection of principle to this. Pragmatically, however, to seek inclusion of every consequence, no matter what the costs are of tracking it down and quantifying it, seems completely over the top: the limits of inclusion ought more rationally to be determined by a sensitivity analysis of whether inclusion or exclusion makes a difference of significant concern to any relevant outcome. At any rate, *reasonably* full information might be conceded as a desirable thing when the objective is to produce a decision that is in the public interest. What constitutes “reasonable” in any given context would necessarily be a matter for local judgment (decision makers and advisers). If the objective were less comprehensive than societal, however, to collect full information would be to collect much that was irrelevant to the context of that decision question. The argument that “more information is always better” is therefore not an argument for adopting the societal perspective; it is an argument for EEs that can reflect many perspectives, including the “societal” one, but only up to the point at the value of further information is judged to be less than the cost of collecting and incorporating it.

What “societal” means is, even in its most comprehensive version stated in the literature, commonly rather more restricted in terms of the nature of the consequences considered than may be thought. Compassionate externalities ^11– 13^ are usually not included. Stresses and welfare losses resulting from changes in delivery pathways, provider identity, loss of employment (temporary or permanent), limited involvement in or being informed about changes, confusion and misunderstanding of the purposes of changes – such effects characteristically accompany both investment decisions and changes in the ways in which decisions are made but none, however, feature in the scope of “societal” as commonly stated. “Societal” is thereby significantly short of being as comprehensive as may appear.

In any event, in practice, not all have followed the Panel’s recommendation. Table 1 provides an illustrative variety of approaches. As would be expected, perspectives offered by an academic do not necessarily reflect the expressed views of public agencies. Developed countries like England, Australia and Canada rely on EE to inform allocation decisions and tend to be narrower in their scope of analysis whereas global Reference Cases tend to adopt a wider perspective. Some low-income and middle-income countries (LMICs), where out-of-pocket costs are significant select a societal perspective though very few have a track record of using EE systematically.

**Table 1.** Recommended perspectives.

Location	Perspective	Comments
Non-governmental organisations		
Pauly (1995) ^18^	Healthcare investments should be evaluated in the same way as any other economic intervention. “The only question is, do they represent a potential Pareto improvement (as measured by individual utility)?”	The perspective of a radical individualist. Great faith in the ability of highly imperfect markets, especially in weakly regulated ones as in low- and middle-income countries (LMICs), to reveal and weight fairly individuals’ willingnesses to pay. Also assumes, even if these willingnesses were truly revealed, that they are ethically appropriate bases for collective choice.
Sanders *et al*. (2016) ^9^	Recommends both a societal and a healthcare system perspective.	Pragmatic but implicitly assumes that EEs are always conducted on behalf of the public interest by a publicly accountable body and that the societal perspective is more comprehensive than it actually is.
Drummond *et al*. (2015) ^19^	Recommends a multi-sectoral perspective because, although it may not embrace all costs and consequences in the economy, it might identify trade-offs of consequence.	Pragmatic, but a better idea might be to spot and roughly assess such trade-off possibilities early in the research commissioning process and then present some bespoke perspectives to the decision maker for their judgment.
Wilkinson *et al*. (2016) ^10^	Like Drummond *et al*. (2015), a disaggregated societal perspective should be used to capture relevant non-health effects and costs	Pragmatic and context-sensitive. Avoids specifying a specific perspective.
IPF Institut für Pharmaökonomische Forschung (2006) ^20^	The choice of perspective must be derived from the research question. The societal-economic perspective is the most comprehensive approach, but other perspectives are possible, e.g. the health system, social insurance, other service providers (hospitals).	Pragmatic but implicitly assumes that EEs are always conducted on behalf of the public interest by a publicly accountable body and that the societal perspective is more comprehensive than it actually is.
International Society for Pharmacoeconomics and Outcomes Research (ISPOR) draft guidance (2017) ^21^	The primary perspective uses costs that fall on the decision maker and QALYs or DALYs as measures of the expected population health impacts including direct effects, herd effects, and serotype replacement. A secondary and broader societal perspective includes costs and effects both inside and outside the health care sector, possibly including educational attainment, productivity, household financial risk, and tourism impact.	Pragmatically generated in specific instances always by the decision problem and whoever “owns” it. The context here is technology-specific - pharmaceuticals in health care. Like others, the possibility that EEs might be usefully deployed by, say private or public hospitals, is implicitly ruled out. Very explicit in advocating QALY or DALY outcome measurement as context-free requirements, which may be impossible in some low-income contexts and not acceptable in others.
World Health Organization (WHO) ^7^	Resource use and health effects should be identified and valued from the societal perspective.	In the case of LMICs this guidance may be hard to implement given informational as well as technical capacity constraints. As with the 2nd Panel’s recommendation always to adopt a societal perspective, it is better for the type of perspective to be determined by the end user rather than the analyst or an external agency. Assumes that the societal perspective is more comprehensive than it actually is.
National governments		
Australia ^22^	Health care sector. A supplementary analysis can be provided using a broader societal perspective.	Pragmatic. Societal perspective a second-line option.
Austria ^23^	A societal perspective, but other perspectives (e.g. health care system, social insurance) are possible.	Like IPF - pragmatic but Implicitly assumes that EEs are always conducted on behalf of the public interest by a publicly accountable body and that the societal perspective is more comprehensive than it actually is.
Belgium ^24^	Costs: Health care payer (government + patients); outcomes: society (for health-related quality of life: health state descriptions by patients, valuations from general public)	Very context-specific, outcomes considered more broadly than costs (as expenditures). Patient preferences specifically required as foundation of outcome measures.
Canada ^25^	In the reference case, the perspective should be that of the publicly funded health care payer. The perspective of the EE should be related to the decision problem. Where perspectives other than the reference case perspective are of interest to the decision-maker and could have a substantial impact on the results of the analysis, these should be included as additional non-reference case analyses. For these analyses, estimate the magnitude of the effects compared with the reference case.	Although the reference case is not wholly context-free, it has a broadly- drawn scope suitable for a public federal organisation with allowances for types of decision problem and for the differing contexts in Canadian provincial health ministries.
England & Wales ^26^	All direct health effects, whether for patients or, when relevant, carers. Include a range of views, especially when there are differences of opinion and a range of patient and carer perspectives, including majority views and views that may be held by only a few patients even if they contradict the majority. The potential impact on resource costs and savings should be considered from the perspective of the NHS and personal social services. In exceptional circumstances, when requested by the Department of Health, the scope will require a broader perspective on costs. This last clause is the main respect in which this guide differs from that issued in 2004.	An example of a country-specific perspective set by the accountable authority (NICE - effectively the Secretary of State for Health). Exceptionally sensitive to receipt of clients’ views. Unusually clear on opportunity costs: “A technology can be considered to be cost-effective if its health benefits are greater than the opportunity costs of programmes displaced to fund the new technology, in the context of a fixed NHS budget”. Also on explicitness: “the Institute has defined a 'reference case' that specifies the methods considered by the Institute to be appropriate ... and consistent with an NHS objective of maximising health gain from limited resources.
Indonesia ^27^	Health technology assessment (HTA) in Indonesia is expected to use societal perspective. The perspective must be expressed either in the protocol and the final report.	The HTA Committee in Indonesia stated in the guideline that the results of an EE will have an impact on a whole society and not on specific organizations or individuals. Therefore, they recommend the societal perspective. The high level of out-of-pocket expenditures in the country may also have been in their minds.
Egypt ^28^	The study perspective should be relevant to the research question and adapted to benefits gained by the health care system.	This is a very flexible recommendation and may reflect the current situation that EE has played minimum role in public resource allocation in Egypt.
Thailand ^29^	Societal and healthcare provider perspectives are both recommended when presenting economic evaluation results to public health authorities.	This recommendation reflects Thai decision makers’ concerns with both out-of-pocket expenditure as well as efficiency of the general tax-financed universal health coverage scheme.

The limits of feasibility are much more tightly drawn in LMICs than in rich countries, where a closer approximation to “full information” may be both possible and desirable. In LMICs, what is possible is much more limited due to poor or absent data, lack of technical skills to conduct and use EEs, and lack of political understanding, leadership and support ^14, 15^. Table 2 shows that a healthcare payer’s perspective is the most popular recommendation in the national guidelines of high-income countries where EE is commonly used to inform reimbursement of health services. Only a third of the guidelines from both high-income countries and LMICs recommend a societal perspective.

**Table 2.** Perspectives recommended by national methodological guidelines.

Perspectives	High-income country guidelines	Low- and middle-income country guidelines	Total
Payer	14(47%)	4(36%)	18(44%)
Health care sector	6(20%)	3(27%)	9(22%)
Societal	10(33%)	4(36%)	14(34%)
Total	30(73%)	11(27%)	41(100%)

Source: GEAR’s guideline comparison http://www.gear4health.com/gear/health-economic-evaluation-guidelines

It would also be rash to suppose that what is regarded as the public interest is always and everywhere the same. Just such a belief underlies the World Health Organization (WHO) advocacy of DALYs as a kind of universal outcome metric with weights attached to dimensions of disability that are invariant with respect to culture and context. This is advanced as an advantage of DALYs over QALYs, since QALYs typically use customized country weights. A similar insensitivity can be seen in the WHO’s abandoned attempts to recommend thresholds ^16^. A threshold is an aggregate expression of collective willingness to pay for treatments in national health insurance. It represents a marginal preference for public expenditure on health care relative to the many other objects of public policy – poverty reduction, education and training, housing, law and order, civil and national defence, and so on. There can be no presumption that one size fits all, regardless of political stability, stage of economic development, culture, religion, or history. We should not encourage a similar insensitivity to enter an authoritative methodological guide to best practice in EE.

## Societal perspective: Whose values and whose judgments count?

A problem with the use of a societal perspective is that it raises the possibility of reintroducing into health policy decisions many of the very biases that most health care systems have been designed to avoid. A critically important one concerns the implications of a perspective for the role of valuation and willingness to pay for health gains. It is part of the conventional wisdom of health economics that the health care market is characterised by more market failures – and serious ones at that – than almost any other market. Many of these impinge on the reasonableness of using individual willingness to pay as a priority-setting criterion and, more generally, of using the Pareto criterion (or the potential Pareto criterion) to identify socially beneficial changes ^17^. These are standard aspects of the textbook treatments of heath economics: we merely list those that are of chief concern ( Table 3). It is not satisfactory, as in Pauly (1995) ^18^ and others who advocate the use of consumer preferences as a basis for public priority setting, to glide over these issues as if they were empirically or politically unimportant. The problems of reliance on consumer preferences for healthcare relate not only to measures of individual (as distinct from collective) willingness to pay but to all expressions of patient preferences. They raise the difficult question of the right balance to be struck between individual expressions of value and collective ones.

**Table 3.** Problems with relying on patient preferences.

Issue	Characteristics and consequences
Socio-economic gradient linking health and wealth	Ill-health and disability are inversely related to ability to pay (income or wealth). At all points on the gradient the better off have better general health and less disability. Willingness to pay is increased by ability to pay so those most in need of healthcare are least able to afford it. Consequently, willingness to pay as a criterion directs resources away from the neediest and will similarly direct collective investments in healthcare technologies ^30, 31^.
Principal-agent discordances	An agent acts on behalf of a principal and is supposed to serve the principal’s interest. A physician’s professional role can become compromised if their private financial interests clash with the patient’s need for medication or with best-practice guidelines. This may be the case with fee-for service systems of physician remuneration, systems in which physicians dispense as well as prescribe, when there is no institutional support for producing and following authoritative clinical guidelines, and where patients’ choices are influenced by biased advertising and inaccurate web-sourced information. These discordances may make patients’ true preferences or willingnesses to pay impossible to detect reliably ^32^.
Supplier-induced demand	A form of principal-agent corruption, in which a demand for ineffective care (such as needless office visits, prescription of ineffective medicines, use of ineffective or harmful surgical procedures) is manufactured by the physician. A consequence is that the value placed on healthcare is exaggerated and promoted by commercially supported patient advocacy groups ^33– 35^.
Asymmetrical information	The most common form of asymmetry is that between physician and patient. Each has knowledge not possessed by the other. Patients are usually experts on their own fears and insecurities, financial and family circumstances, work-related and other social obligations. The professional is usually more expert in diagnosis and understanding treatment options and their health consequences. Problems arise when such knowledge is not communicated and shared – for example the patient’s own expert knowledge of their personal and family circumstances is ignored, or the nurse’s advice and the reasons for it are poorly given, or understood, or not followed (or all three). Other asymmetries can arise, for example between general practitioners and specialists, clinical professionals and managers, though these have a lesser bearing on the revelation of patients’ preferences and values. Asymmetries raise questions about the adequacy of patient preferences, as expressed in their willingness to pay or through surveys, as representations of patients’ true interests ^36, 37^.
Ignorant or prejudiced clinical judgments	Because the demand for healthcare is almost always evidenced through decisions taken by a clinician, poor training or failure to maintain professional standards can result in choices that are not representative of patients’ values or best interests even when the other imperfections are absent. This is especially the case with so-called “complementary” medicine and, in LMICs with “traditional” medicine, little of which is evidence-informed, much of which is unhygienic, some of which is directly harmful, and all of which is conducted in a commercial way. A consequence is that expressed patient preferences need to be interrogated lest they are based on faulty or incorrect clinical understanding ^38^.
Irrational behaviour	The theory underlying the use of willingness to pay as a measure of patient benefit is built upon a set of assumptions about human choice behaviour, all of which have been exposed as false for at least some of the time. The most common (utilitarian) assumptions are: Completeness: either A is preferred to B, or B to A or a person is indifferent between them; Transitivity: if A is preferred or indifferent to B and B is preferred or indifferent to C, then A is preferred or in-different to C; Continuity: there is an indifference curve such that all points to its north-east are preferred to all points to its south-west; Convexity: the marginal rate of substitution is negative; Non-satiation: more is always preferred. If one or more of these axioms is violated the link between choice and welfare is broken and, indeed, the nature of “rationality” becomes ambiguous ^39^.
Patient incompetence	In some circumstances patients are inherently incompetent in whole or part ^40^, typically through illness or disability, youth or old age. In consequence, any value expressed may need careful interpretation.
Externalities	An externality exists when the actions of one person have direct impact on the welfare of another. These may be physical, as when being immunised reduces the risk to others of falling ill (up to herd immunity), or psychic, as when the mere knowledge that poor people have medical coverage increases the welfare of people with sympathetic feelings. A consequence of such effects is that the benefit to the patient underestimates the social benefit (i.e. the sum of the private benefit to the patient and the external benefit to one or more others ^11, 41^.
Public goods	This notion of publicness has nothing to do with the ownership of resources. It refers to the nature of benefit. A public good is one from one from which consumers cannot be excluded (whether or not they pay) ^42^. Classic examples are street lighting and national defence – whether you like it or not you receive the service. Public health measures often have this characteristic: clean water supply and proper waste disposal benefit all (including people downstream who are part of another community). The external benefit of immunisation is generally a public benefit. The theoretically correct way of estimating the social value of such interventions is by summing the value to all who benefit from each scale of operation or, for increases in such interventions, summing the willingness to pay each has for the addition. Apart from the impracticability, this approach is also subject to some of the other objections to the use of willingness to pay. Consequently, an overall judgment has to be made on behalf of the entire community in question

This is not the place to argue the cases for or against individual willingness to pay as a basis for making health investments or of locating the patient as a decision maker in a healthcare system. Our purpose is only to argue that a societal perspective need not necessarily require its source of value, whether or not monetised, to be individual patients. The welfare of patients remains a central concern but avoiding the distortions of the real world is necessary if patients are to be responsibly protected.

What, therefore, is the appropriate source for social valuations of the kind used in EE? And whose willingness to pay for the treatments offered in public insurance benefit packages of the sort aspired to, especially in LMICs, should be embodied in policy? We are not concerned here with individual choices of clinical providers and treatments, though the bulleted issues below do have implications for information provision, clinical audit and health care quality assurance. The questions for EE concern instead the value content of a set of key *collective* judgments that usually have to be made. The answer to all of the following will be shaped by the perspective:

- The choice of outcome measure (life-years? Lives? DALYs? QALYs? Market value of human capital generated, or commercial gain?).
- The construct validity of the possible measures, given the values and culture of country in question (which may themselves be heterogeneous across regions/provinces). (“Does this measure correspond sufficiently to what we collectively understand to be the underlying concept (health)?”)
- The impact of the country’s budget for health care on the affordability of the various potential benefit packages (or extensions to them). (“What would a modest addition to or subtraction from the current level of public expenditure on health and healthcare imply for additional possible interventions, or for those most likely to be removed from the benefits package?”)
- The relative value in real terms (opportunity cost) and at the margin of the various interventions. (“Do we need a money measure or is it sufficient to compare the impact of each on health?”)
- The relative value in real terms (opportunity cost) and at the margin of extensions of entitlement. (“How do we trade-off the benefits of extending the benefits package to more people or specific groups (dwellers in remote places, workers, civil servants, the military...) against increasing the scope and size of the package for existing members of the scheme?”)
- The relative value in real terms (opportunity cost) and at the margin of increased financial protection from the hazard of catastrophic (or significantly large) personal healthcare expenditure. (“Which population groups are most likely to benefit and what is a good measure of that benefit?”)
- The monetary value of an increment in outcome required if an intervention is to be added to the benefit bundle or to displace existing benefits. (“What price adjustments must the manufacturer offer for this intervention to become cost-effective?”)

The collective answers to these questions require no knowledge of individual willingnesses to pay for health care. Instead they require decisions taken at the societal level by decision makers accountable through some locally legitimate political process, advised where appropriate by expert opinion and supported by the best available evidence. Individual preferences/values might be considered relevant in selecting outcome measures, their dimensions (physical, mental functioning, etc.), the weights attached to various dimensions that enable them to be added up to create an index of outcome, and the weights (commonly unity) to attach to individuals when individual levels of health or disability are aggregated to community level. Decision makers need to exercise judgment regarding the extent to which individual members of a community are to be involved in these judgments. The societal judgments that are required are themselves choices that should be context-dependent and essentially locally accountable.

“Societal” does not necessarily imply “everyone”. Decision makers may have their own views about whom to consult and when. They will have their own views as to what exposures to risk and distributions of cost and benefit are fair. They may have general across-the-board financial rules for public sector investments that stipulate discount rates and time horizons. They will address at a higher level what can be afforded for health care in the light of pressing needs for education, housing, poverty relief, law and order, etc. They will decide whether their constitutional preference is to narrow the idea of health to whatever impact health care provision has on it. They will decide whether to consider non-health consequences of health care investments.

## Who are the stakeholders? A tentative list

Stakeholders all have perspectives, some of which may be shared, but most of which will need to be accommodated in some way in the methods of EE and the processes through which it is conducted. The extent to which individual ideas about perspective can be effectively suppressed in the interest of having a shared and publicly stated perspective has not yet, so far as we are aware, been explored empirically. EE analysts can give guidance. For example, a suggested list of possible stakeholders whose roles are for decision makers to consider (which has to be context-sensitive) might be as follows:

- Representative of the general (or relevant regional) public
- Patients with personal or vicarious experience of a relevant condition
- Family informal carers
- Relevant clinical specialists
- Clinical generalists
- Professional leaders (early adopters)
- Politicians (including the opposition parties)
- Researchers (all relevant disciplines)
- Product manufacturers
- Health service providers
- Health service managers
- Aid givers and donor organisations
- Relevant NGOs and other sponsors
- Insurers (public and private)
- Educators
- Media communications specialists and journalists
- Parliamentarians
- The Judiciary
- Other interested parties

Selection of stakeholders and possible roles they might play in determining the perspective are matters on which EE analysts may advise, for example, by listing options, sharing experience elsewhere, indicating likely consequences, and building models.

## On the role of the analyst (and how we differ from others)

Our recommendation is, then, that analysts should approach the issue of perspective at two levels of specificity: the universal or general (context-free) on the one hand, and the context-dependent on the other. A fundamental universal context-free principle is that the perspective always be stated, be consistently adhered to throughout an EE study and appear in reports of the study. Another is that the reasons for the perspective should be given, any departures from previous or common practice elsewhere noted and explained.

These two principles will also shape much of the process in conducting an EE; for example, identifying partners and stakeholders, the extent of consultation, the need for new context-specific research, time-lines and reporting accountability. Their roles will depend upon the context-specific perspective actually adopted. For example, most perspectives will doubtless need to include physicians and patients in consultation meetings, but the scope of the issues discussed will differ according to the specific perspectives: in some studies they might advise on the probable accuracy of the outcome measure and the likely cost consequences of adopting a new technology in the social care sector, in others, they may be concerned only with the outcome measure. The national payer may specify that no additional resources will be available, which will encourage a relatively narrow perspective. Alternatively, the national payer may want an EE performed in order to inform a review of public expenditure across all sectors, in which case a broader public-sector perspective is implied. A health advocacy group, by contrast, may wish a societal perspective to be adopted to establish a case for increasing the health budget. A public payer committed to health maximisation is likely to adopt a perspective that focuses narrowly on health as an outcome while being much broader in its consideration of cost-effective means of promoting health by embracing nutrition, public education, housing and other determinants not normally within the direct control of the Ministry of Health. In each case the perspective is determined by the interests of the client for the analysis. A donor committed to universal health care might adopt any of a number of positions regarding perspective, depending both on its fundamental strategic purposes (perhaps with a single disease perspective) and on the specific context of application (perhaps having specific regard to local capacities to manage complex delivery requirements).

## Conclusions

Our conclusions can be simply stated:

- adopting a societal perspective is not a context-free requirement,
- being explicit about the perspective is a context-free requirement,
- choice of perspective is always and everywhere a matter for the study sponsors to determine together with any stakeholders they select,
- if the sponsors of a study intend it to inform policy decisions (public or otherwise) the decision makers’ perspective should be adopted (along with others deemed to be relevant by the sponsors),
- the role of economists, epidemiologists and the like is to advise, assist, point out likely consequences, and to seek out, adduce, model and interpret evidence,
- the role of economists, epidemiologists and the like is not to engage in advocacy for specific context-dependent perspectives, societal or otherwise.
