# Supplementary material for: Rival perspectives in health technology assessment and other economic evaluations for investing in global and national health. Who decides? Who pays?
Source: F1000Res. 2018 Jan 17;7:72. [Version 1] doi: 10.12688/f1000research.13284.1 (PMC5961761; doi:10.12688/f1000research.13284.1)
Supplement: Peer review history

# Peer review: Referee response for version 1

**DOI:** 10.5256/f1000research.14416.r30070

**Published:** 2018-03-07

**Authors:** Carol Levin

**Affiliations:** 1 Department of Global Health, University of Washington, Seattle, WA, USA

As a practitioner who conducts costs and cost-effectiveness analysis in low and middle-income countries, this was a useful paper and reminder to consider context and include stakeholder input early in the process as best practice. I appreciated the history of perspective and the clear conclusions simply stated.  I have replied partly to two of reviewer questions because I read this opinion paper through the lens of a practitioner working only in a global health context, with familiarity of the published papers across a number of global health conditions. 

 As I read the paper, I wondered who the audience is for this paper? Is it researchers in high-income countries? Is it intended for researchers in global health economics? Is the purpose of the paper to counter recent recommendations of the second panel on cost-effectiveness or the second “Washington Panel”?

 I wasn’t quite sure what the problem being addressed is and who the intended audience for this paper was, since it combined a mix of practical points, theory and recommendations.

 Recommendation: In the introduction, can the authors add a problem statement? And then explicitly indicate if these recommendations are for researchers in high-income countries or global health settings or both.

 Also, as a practitioner, my experience is that analysts, conducting global health economic evaluations, more often than not, use common sense and already use a provider or (provider and societal perspective) in the majority of published economic evaluations in global health (whether for evaluating health technology, vaccines, diagnostics, medicines, delivery strategies). For instance, I selected two annexes available through the *Disease Control Priorities Project* for Reproductive Maternal and Child Health CEA studies and for Cardiovascular CEA studies and found that out of 295 articles, 22% were conducted from a societal perspective and 52% were from a provider perspective and 15% were from both. For CVD prevention or treatment CEA studies, 27% were from a societal perspective and 68% from a provider perspective. 

 This may not be the same as more deliberately using context specific perspective. So this either means that researchers conducting global health economic evaluations are already pragmatic, engaging stakeholders to select a context specific perspective, or that they are not following recommended guidelines. The authors note that only a third of the guidelines from both HIC and LMIC recommend a societal perspective. That certainly makes sense to me, for LMIC where economic evaluations are often conducted to introduce a new health technology or delivery strategy, where the status quo is nothing (or very low coverage of existing technologies or services).

 As the authors nicely present, EE was method first developed for use in high-income countries, with a very specific objective in mind. They were not developed with global health interventions and strained governance and health systems in mind. However, as interest and capacity grows in LMIC to conduct EE, guidelines are extremely useful for researchers at nascent stages of learning a new method. And these guidelines typically get adapted initially and over time to better suit applications in global health, through disease specific recommendations/guidance/guidelines promoted by WHO or other multi-lateral or global health organizations. 

 On page 4, the authors note that there can be no presumption that one size fits all, and states we should not encourage a similar intensity to enter an ‘authoritative methodological guide to best practice in EE’. I’m not sure that analysts DO presume a one size fits all. This is very clear from any review of a set of CEA studies that look at exactly the same two alternative strategies to address the same health condition.  Comparability across many studies is difficult. I’m also not sure whom the authors are referring to and if there is a problem among researchers conducting global health EE with respect following authoritative guidelines ( *There are many problems in standard methods and reporting across studies—and these do cause a problem for using resulting study results—suggesting that guidelines are important to improve quality and comparability of studies—even when appearing authoritative—not sure what is wrong with that when talking about methods)*. When the guidance is not feasible or even does not make sense (i.e. using a societal perspective), it does seem that these recommendations are simply not followed. This may explain why provider perspective is used more often than provider perspective in EEs in LMIC.

 Recommendation: Is it possible to take into consideration what is done in practice and acknowledge that practice already is very different from the guidance—certainly in LMIC?

 Recommendation: On page 3, column 1, par 2, suggest indicating that guides to best practice can be traced back at least as far as 1980 **developed for use in high income countries to inform government allocation of resources (or something to this effect). **Very important to note these guidelines were NOT initially developed for LMIC, but adapted more recently for that purpose.

 Quality and comparability in economic evaluations is challenging. In the introduction, the authors recognize the need to set international quality standards, but I take some issue in calling those that follow the standards falling into a “trap of small-town thinking”. Perhaps this is not what the authors meant, but I’m not sure that it is parochialism at work, but rather a desire to generate comparable results, using economic theory as a foundation for the recommendation. Perhaps it is this underlying theory that the authors take issue with.

 The authors understand the history and economic theory underlying the recommendation of a societal perspective much better than I, but it is unclear to me what the economic debate is and between who? They allocate a section in the paper to a theoretical discussion on the issue of individual expressions of value and collective ones. While I found this interesting, it seemed somewhat out of place to me, if this paper’s audience is practitioners in low and middle-income countries, doing their best to figure out the best way to conduct economic evaluations. 

 Recommendation: What is the consequence of wrongly using a societal perspective? Can the authors more simply explain this to readers in the introduction or somewhere in the paper why a societal perspective may not allocate resource efficiently and equitably? 

 This gets me to my final comment on the paper, which it is a mix of a thought paper and a recommendation or guidance paper. I found myself wondering about the ‘how’ on page 9 when authors suggest engaging a long list of stakeholders in determining the perspective of the EE analysis. I agree with this, but can the authors refer the reader or add information on how to do this? This becomes particularly important for academics that are conducting EE in LMIC without direct access to stakeholders. This is often the case for researchers conducting EE for a specific health technology (vaccines, drugs, diagnostic, mhealth application,) that is under development. In addition, even when new services or technologies are funded and ready for introduction in LMIC, demand comes from the donor, researcher or NGO, not the in-country stakeholders that will ultimately introduce and fund the health intervention. However, I fully agree, when stakeholders are involved, identifying who will USE the information, and engaging them early makes it much easier to determine the objective and perspective of the study.

**Minor comment**

**Abstract**

 1. Suggest that the authors provide a main recommendation in the abstract. Or end the abstract with a more positive statement stating that economic evaluation becomes more relevant when the perspective is sensitive to context and appropriate for the objective of the analysis.

I have read this submission. I believe that I have an appropriate level of expertise to confirm that it is of an acceptable scientific standard, however I have significant reservations, as outlined above.

# Response

**Published:** 2018-03-21

**Authors:** Benjarin Santatiwongchai

The authors are grateful to the reviewers for the evident care they have taken in reviewing our paper.

Carol Levin requests a problem statement and greater clarity as to its intended readership. We thought we had stated the “problem” clearly in the Introduction, namely that what we called “small-town thinking” imposes arbitrary limits on the perspective from which economic evaluations are conducted. Furthermore, we do not regard it as the business of analysts (when acting as analysts) to assert appropriate perspectives. It follows that, since this is a point about scientific method, our article is addressed at one level to methodologists, textbook writers and others who seek to provide training materials and set standards of good practice for economic evaluations. Since good practice is something that practitioners ought to follow, practitioners need to be alerted to the possibility that not all expert recommendations on good practice may be sound. While this is a perfectly general point, which requires us all to be critically alert to the possibility of poor guidance, we quite specifically aimed a criticism at the common recommendation that studies be conducted from the “societal” perspective. We offered a little history of this approach, which has begun to assume the status of a common standard.  The main reason for our view is that to select a perspective (any perspective) is to make a social value judgment about what “counts” in designing and implementing public policy, including health policy. The perspective defines the benefits, costs and harms that are to be considered as well as the ways in which they are to be brought into a single judgment about the eligibility of clinical procedures for inclusion in an insurance (public or private) scheme. Analysts are not empowered (qua analysts) to make these judgments. Those who *are* empowered include the managers of private health insurance schemes (typically accountable to a board, to trustees, or to shareholders) and the managers of public schemes, usually accountable to a minister and through the minister to an electorate. These people are commonly referred to as “decision makers”. This accountability does not apply to analysts – unless of course they are themselves also managers, trustees, etc. Accordingly, good practice for analysts is to assist decision makers in selecting a perspective that is defensible and in accordance with the social and political values the decision makers in question are expected to live up to.

One way in which this can be done is to have the decision makers endorse a formal methodological approach that should characterise all the analysis commissioned for that specific jurisdiction. This is very common amongst jurisdictions that have set up specialist agencies to make decisions (like NICE) or to make recommendations (like HITAP or CADTH). We observed in our review that the societal perspective does not usually characterise these guides to method, or “reference cases”. Nor are they by any means identical, despite having been devised with assistance of evidently competent economic analysts. This should not be a matter for regret. Rather, the presumption is that each has been designed for the particular circumstances of the jurisdiction to which it applies. Amongst other implications is that academics conducting analyses “without direct access to stakeholders”, as Dr Levin puts it, are at great risk of producing recommendations that are irrelevant in any specific context. Good practice does not invariably require huge amounts of interaction with stakeholders but when the client for analysis has made no statement about perspective, and stakeholders are beyond reach, good practice is most likely to be “no practice”. For whom, one might ask, might such an uninformed analysis be intended?

# Peer review: Referee response for version 1

**DOI:** 10.5256/f1000research.14416.r29943

**Published:** 2018-01-24

**Authors:** Alexander Winch

**Affiliations:** 1 PRICELESS SA (Priority Cost Effective Lessons for System Strengthening South Africa), University of the Witwatersrand, Johannesburg, South Africa

**Is the topic of the opinion article discussed accurately in the context of the current literature?**

- This is a welcome and much needed addition to the dialogue on improving the quality of conducting and reporting economic evaluations and HTA internationally.
- Whilst the linkage is made well between the need for an explicit perspective as part of a robust high quality economic evaluation, one opportunity could be to explicitly state the importance of a well defined and robust decision problem as a fundamental to being able to define an appropriate perspective for an EE. Whilst this may be clear for those familiar with the guidelines, however the intended audience may not be familiar with the concept of scoped decision problems.
- The section on the history of perspectives in methodological guidelines for conduction EE covers well the main evolution in guidelines. One potential enhancement could be through a discussion on why it took 20+ years for the perspective of an EE to be included explicitly as a characteristic in guidelines.
- The article is clear on the justification for an explicit perspective, and why broader is not always better when it comes to 'societal perspective'. However, one opportunity for the article could be to look at the misuse or misunderstanding of concepts and language between policy makers and the technical EE analysts when it comes to the perspective on an evaluation.
  For example, policy decision makers may be supportive of using a 'societal perspective' for an economic evaluation in a context for which this may not be appropriate, yet actually be conflating this concept of the 'societal perspective' with undertaking an inclusive, stakeholder engagement strategy in the health decision making process.
  This conflation, is similar to the use by policy decision makers of terms such as 'cost-effective' of an intervention, even when this is not directly linked to the concept of opportunity cost, or the use of a context specific CE threshold, creating as a weird consequence the dichotomy of something being deemed 'cost-effective' yet 'unaffordable'.

**Are all factual statements correct and adequately supported by citations?**

- All factual statements are correct and supported by citations.
- Paragraph 7 could strengthen the assertion that ‘Some LMICs, where out-of-pocket costs are significant select a societal perspective though very few LMICs have a track record of using EE systematically’ through references to LMICs with high OOPs, or LMICs track record of EE use in decision making

**Are arguments sufficiently supported by evidence from the published literature?**

- All arguments made in the article are supported by evidence from the published literature.
- In paragraph 3, the assertion that 'a **good **study will be, therefore, explicit about its perspective’ could be enhanced through using terms such as 'high quality', 'economically rigorous' or 'comprehensive' as this can then be referenced back to the principles of the iDSI reference case.

**Are the conclusions drawn balanced and justified on the basis of the presented arguments?**

- The conclusions drawn are balanced and justified on the basis of presented arguments.
- Dependent on the intended audience for the article, the language could be simplified from 'context-free' and 'context-dependent' requirements, and instead restructure the conclusions for both the EE analyst (e.g. 1. advise, assist and point out likely consequences to decision maker 2) Not to engage in advocacy of any single perspective), and conclusions for the policy decision maker (1. adopt an explicit perspective in an EE 2. Be explicit in the reasons behind the choice of the perspective etc..)

**Minor additional comments: **

- Table 2 displays payer, health care sector, societal as discrete categories – Does this double count those countries where multiple perspectives may be recommended in guidelines? Or are they given one discrete category? – For clarity it might be good to include a 'multiple' category to the table
- For standardization and clarity, the language under column 'perspective' in Table 1 could be greater aligned. E.g:
  ‘Costs: Health care payer (government + patients); outcomes: society’
  ‘Health care sector….’
  ‘In the reference case, the perspective should be that of the publicly funded health care payer.’
  ‘Health technology assessment (HTA) in Indonesia is expected to use societal perspective.’
- Last bullet point on conclusions is repeated

I have read this submission. I believe that I have an appropriate level of expertise to confirm that it is of an acceptable scientific standard.

# Response

**Published:** 2018-03-21

**Authors:** Benjarin Santatiwongchai

The authors have much sympathy with Alex Winch’s comment on the ambiguity of language and the very real possibility that the understanding of decision makers and technical experts may be significantly divergent. This is probably something that can only be addressed through training (especially the training of health economists) and through careful explaining and re-explaining. It is not helped by devising new names for economic evaluation (currently running at around 24 near synonyms) or by inconsistent use of definitions even amongst economists (a spectacular demonstration of such disagreement about “opportunity cost” is Ferraro and Taylor 2005, but much the same embarrassing variation exists concerning “comparative and absolute advantage”, “cost-effectiveness”, “efficiency” or “equity”).

Ferraro PJ, Taylor LO (2005) Do economists recognize an opportunity cost when they see one? A dismal performance from the dismal science, Contributions in Economic Analysis & Policy, 4. doi:10.2202/1538-0645.1469.

# Peer review: Referee response for version 1

**DOI:** 10.5256/f1000research.14416.r29941

**Published:** 2018-01-24

**Authors:** David Tordrup

**Affiliations:** 1 Triangulate Health Ltd, London, UK; 2 WHO Collaborating Centre for Pharmaceutical Policy and Regulation, University of Utrecht, Utrecht, Netherlands

The authors present a historical review of the origin of "perspective" in economic evaluation, and proceed to critique the standardisation of perspective, proposing that it is inherently context-specific. The paper is interesting and well written. I would make the following points:

1. If we accept that guidelines for EE written by e.g. Drummond *et al.* are intended for evaluation of healthcare interventions, with the decision problem being to adopt or not adopt a new technology, and that these guidelines are at least implicitly developed with the UK context in mind, this means that - yes - the guidelines are intended to generalise away from a specific context (the technology) - but - they also generalise within a specific context (the health system). The same can be said for methods guidance published by NICE. So there are situations where the end users of evidence are issuing guidance on the approach to EE generation, to determine best practices and what evidence is acceptable to the decision problem. The authors concede this, I believe, when they write in conclusion "if the sponsors of a study intend it to inform policy decisions (public or otherwise) the decision makers’ perspectives should be adopted (along with others deemed to be relevant by the sponsors)". This should mean that not all standardisation of perspective is necessarily bad.
2. Further to point 1, if the authors believe that each EE study intended to inform a public healthcare payer perspective should have its perspective built from scratch, how would the consumer of the evidence (the payer) avoid "cherry picking" by the sponsor (a manufacturer) of the most economically advantageous outcomes of the new technology under consideration? And if the perspective should not be built from scratch, how is that different from accepting to have some form of standardised guidance on perspective?
3. The authors also note capacity as a constraint in LMIC's, it seems point 2 would pose an even more substantial risk of "evidence capture" in such settings, where the majority of resource and technical capacity could easily be on the sponsor side, and not the evidence user side. In such cases, the capacity of the evidence user to articulate their needs could be severely limited.

So, while I fully agree that the choice of perspective should be determined by the user of the evidence, I think the authors should revisit their arguments against guidelines on perspective with the points above in mind. Are there cases where it is desirable to have standardised perspective guidance? What are the situations where it is undesirable - presumably where it is simply the result of asserting academic authority or other vested interest?

I have read this submission. I believe that I have an appropriate level of expertise to confirm that it is of an acceptable scientific standard, however I have significant reservations, as outlined above.

# Response

**Published:** 2018-03-21

**Authors:** Benjarin Santatiwongchai

In response to David Tordrup, we should emphasise that we neither said nor implied that a “standard” perspective is ”bad”. The question is: who chooses the perspective? NICE, CADTH and HITAP each has a standard perspective that is fit for its local purpose and chosen by those accountable for making decisions or recommendations in that context. Each of the authors of this paper has been both analyst and decision maker (the latter as the employee or non-executive officer of an HTA agency}. Our accountabilities in each role are not the same. In particular, our skills as scientists do not qualify us to make value judgments on behalf of society. At the most, the kind of value judgments we are entitled to make are value judgments about the quality of the science, which are certainly judgments of value, whether the science is good science, but they are not judgments about what is good for society. What qualified us as makers of social value judgments was our appointment through a legitimate process.

Our view on perspective does not involve every study building a perspective from scratch. On the contrary, there is much to be said for establishing agencies like NICE etc. which are competent to set standards appropriate to their local context. In many cases analysts might usefully be employed in helping to build such a standard “from scratch” but, once set up, the work becomes one of maintenance as new techniques of analysis emerge, or new data, or new criteria are suggested. As far as any specific study is concerned, its authors should follow the guidance as set out by decision makers or else guess as best they may what the perspective might be of those whose decisions they seek to influence.
